# Microfluidic perspectives on chimeric antigen receptor T-cell migration in solid tumours: highlighting physical confinement as a key barrier

**DOI:** 10.1039/d5me00232j

**Published:** 2026-05-15

**Authors:** Valeria Gonzalez Abrego, Matthew H. W. Chin, Marc-Olivier Coppens

**Affiliations:** a Centre for Nature-Inspired Engineering & Department of Chemical Engineering, University College London Torrington Place London WC1E 7JE UK v.abrego@ucl.ac.uk matthew.chin.15@ucl.ac.uk m.coppens@ucl.ac.uk

## Abstract

Chimeric antigen receptor (CAR) T-cell migration and infiltration into solid tumours remains a central challenge for cancer immunotherapy. While factors such as chemokine gradients, extracellular matrix (ECM) stiffness, and immunosuppressive signalling have been well-documented, the physical confinement imposed by tumour architecture is increasingly recognized as a critical barrier. Tumour-associated ECM remodelling generates micro- and nano-scale geometric constraints that significantly alter how immune cells, particularly CAR T-cells, navigate the tumour microenvironment (TME). This article argues that physical confinement merits to be positioned at the centre of the migration challenge, not as an accessory to chemical or biochemical cues. Sustained from both fundamental cell migration biology and recent engineering advances, we highlight how designing microfluidic devices with defined geometric features can uniquely capture the role of confinement in immune cell behaviour and model this challenge. By enabling precise design control over pore size, channel geometry, and nature-inspired structures, these platforms will allow researchers to decouple confinement from other overlapping variables such as stiffness.

## Abstract 2

This perspective identifies physical confinement as a primary CAR T-cell migration barrier and proposes microfluidics to study infiltration and improve therapeutic outcome.

## Graphical abstract

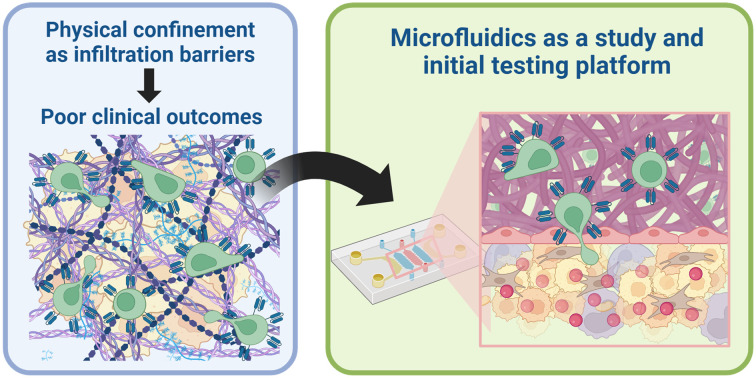

**Design, System, Application**

Chimeric antigen receptor (CAR) T-cell therapy is significantly limited in solid tumours due to poor infiltration into the dense tumour microenvironment (TME). This perspective centres on positioning physical confinement, the geometric constraint imposed by the TME's micro-architecture, as a critical, yet underappreciated, constraint for CAR modified cell efficacy. We discuss the necessity of advanced testing systems and propose the design of microfluidic platforms as a precision tool to replicate this biophysical challenge. By designing and incorporating geometric constraints, these systems could decouple confinement from other cues (*e.g.*, chemokines and stiffness) and help us understand certain limitations of immunotherapy. These integrated multi-parametric microfluidic platforms should become essential systems to validate engineered CAR T-cells, not only by measuring their cytotoxicity efficacy, but also by allowing for quantitative measurement of the effect of mechanical stress on cell health, migration speed, and metabolic exhaustion. Designing these types of platforms can bring us one step closer to engineering CAR T-cells that are physically able to achieve superior clinical outcomes.

## 1. Introduction: the solid tumour fortress

Chimeric antigen receptor (CAR) T-cell therapies have truly revolutionised the treatment of liquid cancers, such as leukaemias and lymphomas, resulting in high response rates and durable remissions.^1^ Against solid tumours, however, CAR T-cell therapy has not been as effective.

While there are multiple reasons, a primary factor is the profound hostility of the solid tumour microenvironment (TME). The TME is an intricate, evolving ecosystem involving a dense, remodelled extracellular matrix (ECM), suppressive stromal cells, like cancer-associated fibroblasts (CAFs), dysfunctional vasculature, and numerous inhibitory biochemical signals.^2^ This represents a significant barrier affecting CAR T-cells, as well as other adoptive cell therapy modalities, including CAR natural killer (NK) cells.^3^

**Fig. 1:**
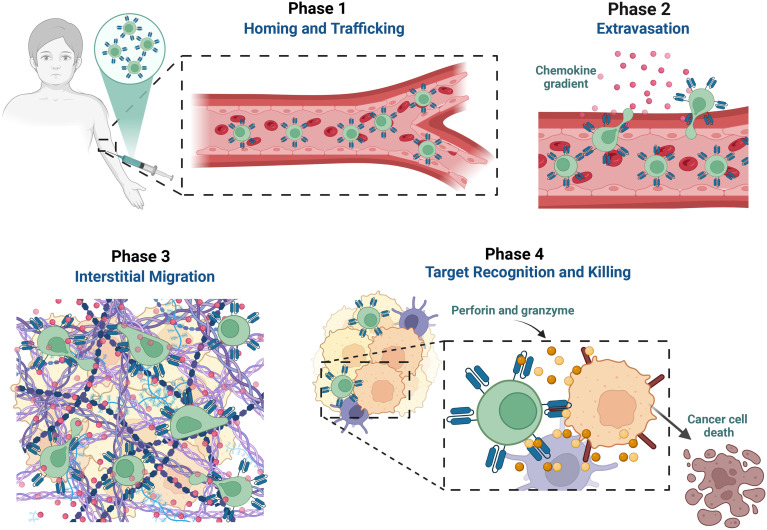
The multi-phase cascade of CAR T-cell migration into the solid tumour microenvironment. Phase 1 involves cells localizing the tumour site, driven by regional chemokine gradients. Phase 2 requires the cells to transmigrate across the vascular endothelial barrier and into the perivascular space. Phase 3 is the critical stage where cells must migrate through the stiff and dense ECM. Phase 4 only occurs if the immune cell successfully overcomes the prior physical and functional barriers and retains sufficient energy to execute its cytotoxic function. Failure in phase 3 is argued to be a central, rate-limiting determinant of the outcome of CAR T-cell efficacy in solid tumours.

For cell-based immunotherapy to be effective, the infused cells must execute a multi-step infiltration cascade: circulate through the bloodstream, exit the vasculature (extravasation), and, most critically, migrate through this dense stromal maze to reach and kill its target cancer cell (Fig. 1). Here, this interstitial migration step is where infiltration often fails. To date, research into this failure has largely focused on biochemical barriers, such as suppressive signals (*e.g.*, PD-L1 and TGF-β) and insufficient chemokine gradients,^4–8^ metabolic barriers like hypoxia-induced immune exhaustion,^9–12^ or bulk mechanical barriers, such as a desmoplastic ECM.^4,5,9,10,13–15^

However, a distinct and critical barrier exists, one rooted in physics and geometry that must be addressed, primarily, when designing cell-based immunotherapy mechanisms and assessing their performance. The TME's dense, fibrous ECM, rich in collagen, is not just stiff, it also forms a micro-structured environment with pores and channels often smaller than the diameter of a T-cell itself.^16^ This article argues that this physical infiltration problem, known as physical confinement, should be incorporated into the design of current testing models as a primary checkpoint. A CAR T-cell that cannot physically migrate through the ECM to reach the tumour cannot be activated, kill its target, or succumb to metabolic exhaustion in the tumour core.

This presents a classic system design challenge. In traditional *in vivo* or 3D bulk hydrogel models, all these variables are entangled. A denser collagen gel, for instance, is not only stiffer but also has smaller pores and different ligand spacing. It is complicated to isolate which cue is a dominant barrier. By using high-precision techniques like 3D microprinting and microfluidic devices, studies can move beyond abstract models and reconstruct the ECM's physical architecture to study and observe the capability of immune cells to migrate and the challenges they present while attempting to infiltrate geometries like the ones found in solid tumours. Microfluidic systems, through their inherent design precision, offer a powerful solution to model this fortress, barrier by barrier.

## 2. Deconstructing the fortress: microfluidic designs for TME barriers

**Fig. 2:**
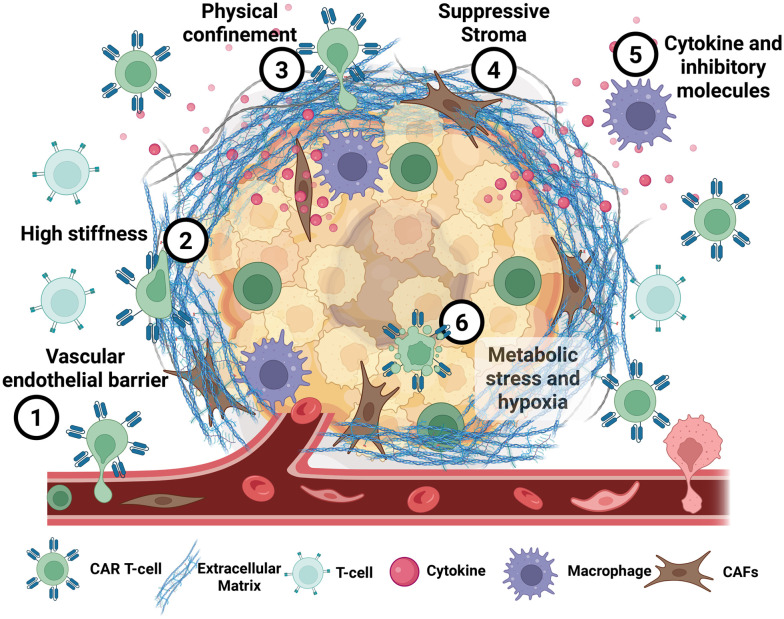
Migration challenges found in the solid tumour microenvironment. This schematic details the six primary barriers detailed in this work that impede CAR T-cell infiltration and function within the TME.

The strength of microfluidic engineering lies in its ability to build bio-inspired systems allowing the isolation and study of individual components of the TME. The challenges to infiltration (shown in Fig. 2) can be broadly classified into two categories. The first are physical and structural barriers, which comprise the physical impediments to immune cell movement: the vascular endothelial barrier (challenge 1) and the dense, stiff ECM (challenge 2), which creates the geometric physical confinement (challenge 3). The second are functional and immunosuppressive barriers, which actively modulate immune cell viability and function, such as the presence of a suppressive stroma (challenge 4), the complex gradients of chemokines and inhibitory molecules (challenge 5), and the harsh metabolic stress from hypoxia and nutrient depletion (challenge 6).

**Fig. 3:**
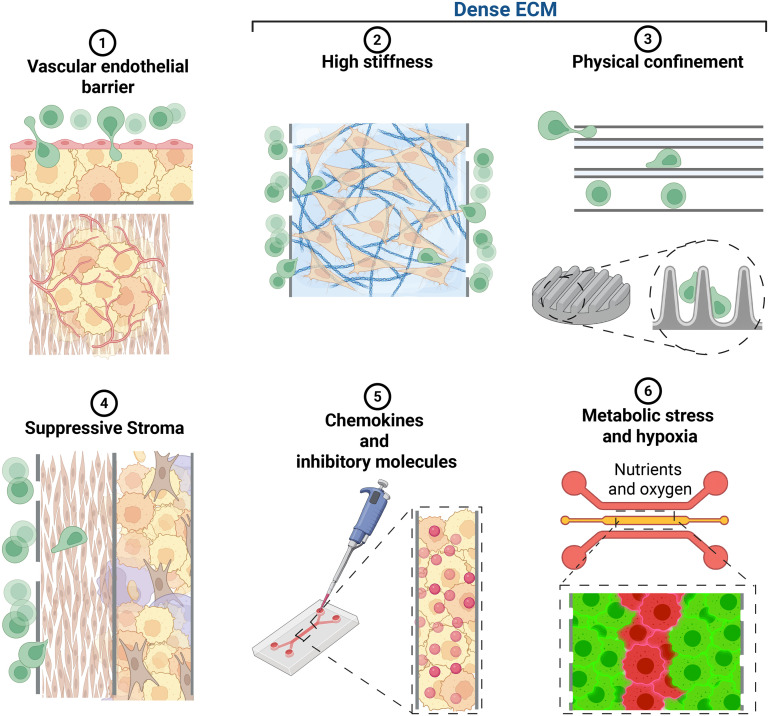
Microfluidic design strategies to model key TME barriers for immunotherapy cell studies. Illustration of the core strategies used in microfluidics to decouple TME migration challenges. (1) Vascular endothelial barriers are modelled by microchannels lined with endothelial cells; spontaneous vessel formation is another way of modelling this challenge. (2) High stiffness is often modelled by encapsulated tumour cells in hydrogels of defined stiffness. (3) Physical confinement has been modelled with micro-channels or pillar arrays. To consider (4) suppressive stroma, co-culture systems using separate or the same compartments have been widely utilized. The incorporation of (5) chemokines and inhibitory molecules is frequent in gradient generators to maintain stable chemical fields to study chemotaxis effects. (6) Metabolic stress and hypoxia have been modelled using multi-layer chips with controlled fluid flow to establish physiological oxygen and nutrient gradients.

Prior to a specific focus on confinement (challenge 3), it is useful to examine how micro-platforms have been engineered to deconstruct and model these other key barriers. Fig. 3 presents a schematic of how microfluidic design has replicated said challenges.

The first structural challenge is the vascular endothelial barrier (challenge 1). CAR T-cells must first exit the bloodstream *via* extravasation, fighting the mechanical pressure small vessels can exert upon them. This is modelled in current literature using tumour-on-a-chip devices that feature microchannels or chambers lined with endothelial cells, forming a perfusable, vessel-like lumen. T-cells can be introduced under physiological flow, allowing for quantitative, real-time observation of their adhesion, rolling, and migration across the endothelial barrier into a tumour-mimetic culture or a cell embedded hydrogel.^17–23^ Other models have also shown spontaneous vessel formation when coculturing tumour spheroids with endothelial cells.^4,24^

Once outside the vasculature, T-cells encounter a stiff and dense ECM (challenge 2). The TME is particularly fibrotic and stiff, aspects which influence migration *via* durotaxis.^25^ This has been previously modelled by integrating tuneable hydrogels, such as polyacrylamide (PAA), GelMA or collagen, into microfluidic devices. By controlling the crosslinker or gel precursor concentration, these platforms present T-cells with substrates of varying stiffness (*e.g.*, from soft 2.3 kPa to rigid 1000 kPa) and highlight how T-cell migration speed drops significantly on stiffer matrices.^13,26–29^ Apart from migration speeds, other studies have also shown how other immune cells, in this case NK cells, change the migration mechanism when exposed to confinement in bulk hydrogels by alternating between periods of random movement, directed migration and migration arrest,^30^ highlighting the importance of understanding migration patterns.

Beyond these physical hurdles, immune cells encounter also functional barriers. Navigating the matrix involves responding to chemokines and inhibitory molecules (challenge 5). The TME is a complex mix of both attractant chemokines (like CXCL12) and inhibitory cytokines (like TGF-β) or exhaustion signals (like PD-L1). Microfluidic devices, with their precise fluidic control, are ideal for this challenge. They can generate stable, long-range chemical gradients that mimic *in vivo* signalling, moving far beyond simple transwell assays to study how T-cells make decisions when faced with competing guidance cues.^8,9,31,32^

These signals are produced by the suppressive stroma itself (challenge 4). The TME is inhabited by a multicellular population. Cells like CAFs, M2 macrophages, and pancreatic stellate cells (PSCs) actively create an immune-excluding barrier that decreases the efficacy of immune cell migration. A microfluidic system model might integrate these signals as chemical gradients or use a multi-chamber system with different cell types or co-cultures. These designs physically separate T-cells from stromal and cancer cells or place them in direct contact, allowing researchers to parse the effects of secreted paracrine signals *versus* direct cell–cell contact. Such models have been helpful in showing how CAFs not only secrete suppressive factors but also actively build a dense ECM barrier, physically excluding T-cells.^4,10,15,17,32^

Finally, the entire TME is subject to metabolic stress and hypoxia (challenge 6). Due to poor vasculature and high cell density, the tumour core is often hypoxic, nutrient-deprived, and acidic. Microfluidic devices can replicate this in several ways, *e.g.*, by designing devices with integrated gas channels for precise oxygen control, or by leveraging the self-generation of oxygen gradients by densely packed spheroids within a chip. These models allow for the study of CAR T-cell migration and function under defined metabolic stress, revealing, for example, that hypoxia can suppress cytotoxicity.^7,9,11^

Such models have provided immense insight into the individual components of TME resistance. However, they also highlight a critical, unresolved design problem: in many ECM-inspired models, the variables of stiffness and density (challenge 2) are conflated with the geometry of the matrix, or physical confinement (challenge 3). As mentioned, a stiffer or denser collagen gel might also be a more confined one, with smaller pores. Although existing methods such as interpenetrating polymer networks (IPN) gels have been implemented to address this problem,^33,34^ to truly understand the infiltration barrier, we must move from bulk gel models with intertwined parameters to designing systems that incorporate a predetermined geometry.

## 3. The challenge: isolating physical confinement by design

While significant progress has been made in modelling the biochemical and bulk mechanical aspects of the TME, replicating the geometric constraints of physical confinement remains a distinct and tough challenge. This difficulty arises from the nano/micrometre-scale complexity of the ECM's fibrous architecture, which is poorly recapitulated by bulk hydrogels. It is precisely at this intersection of complex architecture and cellular-scale physics that design and engineering offer complementing set of tools. By leveraging microfabrication techniques, such as photolithography, soft lithography, or 3D nanoprinting, we now have the capability to design, fabricate and model ECM-inspired features with high precision. These techniques allow for the high-precision fabrication of not only simple, defined geometries, such as channels, pillars, and obstacle arrays, but also more complex and bespoke micro-architectures designed to recapitulate specific features of the TME. This design capability provides a direct way to systematically observe cellular behaviour when the only challenge is physical confinement.

Although scarce, results from platforms modelling physical confinement have highlighted the role of the TME in immune cell infiltration. Studies using microchannels of decreasing widths (from 10 μm down to 2 μm) have revealed a critical size threshold; while T-cells migrate efficiently in wide channels, their migration speed drops significantly as the channel narrows to below ∼3–5 μm, approaching the diameter of the T-cell nucleus.^16,35,36^ This non-linear drop in speed is attributed to the nuclear bottleneck; the nucleus, being the largest and stiffest organelle, must be deformed at a significant expenditure of time and energy to pass the constriction. This migration mode is not only slow but can also lead to nuclear envelope rupture and DNA damage.^37^ Recent comparisons in similar microchannel environments suggest that NK cells may possess a mechanical advantage over T-cells, due to the better deformability of their nucleus. A study demonstrated that NK cells exhibit a higher invasion potential in dense, patient-derived tissues, likely driven by superior nuclear flexibility that allows them to navigate tight physical constraints more effectively than their T-cell counterparts,^38^ further supporting the argument that nuclear deformation actively affects immune cells' migration mechanism and infiltration success.

Most critically, this physical barrier may reveal an intrinsic defect in CAR-T cells, which may be affected more than other immune cells. A recent study by Zhang-Zhou *et al.* (2025) directly compared T lymphocytes and CAR T-cells in a confinement device, finding that CAR T-cells were more affected by mechanical constraints and less efficient at navigating confined spaces.^16^ This suggests that the act of engineering a T-cell may alter its biophysical properties, inadvertently making it a poorer physical infiltrator. This physical deformation also has a metabolic cost. The same study noted that CAR T-cells presented higher mitochondrial activity while migrating than T-cells, peaking under severe (2 μm) confinement.^16^

This strongly implies that these cells are burning critical energy reserves just to move, long before they ever reach a cancer cell to perform their cytotoxic function. Therefore, physical confinement is not just another barrier but a gatekeeper and draining mechanism that needs to be modelled and studied in detail. Confinement has shown to increase the energetic cost of migration, making physical constraints a direct regulator of the metabolic burden placed on migrating cells.^39^ In this sense, the “collagen-maze” dictates if a CAR T-cell can arrive at its target and suggests that, if it does, it arrives already metabolically depleted and physically stressed.^16^ Recent work has shown how cytotoxic T-cells' migration in 3D environments improves when mitochondrial function is enhanced,^40^ reinforcing the concept that metabolic vulnerability is tightly coupled to T-cell persistence, and supporting that mechanically demanding processes such as confined migration may exacerbate this metabolic fragility even before tumour engagement.^41^

## 4. Limitations and the need for multi-parametric systems

**Fig. 4:**
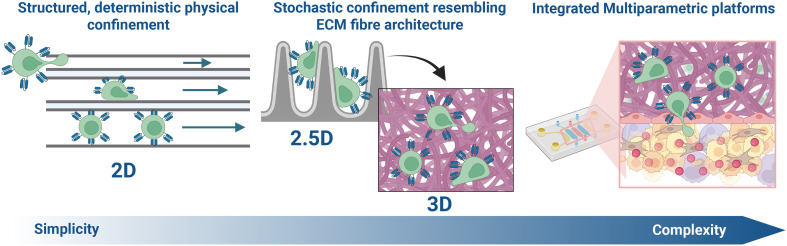
The evolution of microfluidic models toward modelling complexity. Progression from deterministic, reductionist microfluidic confinement models to stochastic, ECM-inspired architectures and finally to multi-parametric systems integrating physical, biochemical, and mechanical barriers. This evolution reflects the need to transition from isolating individual variables to embracing engineered complexity in order to capture inherent behaviours that govern CAR T-cell low efficacy in solid tumours.

The deterministic, structured, single-variable models described in the previous section are already insightful. They represent a classic reductionist approach, which allow us to deconstruct the TME and isolate the specific contribution of physical confinement. However, this simplification is also their primary limitation. The real TME is not a clean, 2D array of parallel channels or obstacle bars but a 3D, tortuous, and stochastic maze of collagen fibres. As has been argued in the context of immunotherapy, a purely reductionist view can be misleading, and there is a need to move towards embracing engineering complexity.^42^ Therefore, the path forward is not to abandon these simplified models, but to use them as building blocks for more complex, multi-parametric systems that significantly represent the interplay of the TME's physical barriers, as illustrated in Fig. 4.

This design approach highlights clear engineering opportunities. A primary need is the transition from 2.5D to 3D; many pillar and channel arrays are fundamentally only 2.5D (*i.e.*, cells migrate on a 2D surface with obstacles in an effort to incorporate a third dimension), whereas true 3D confinement models, achievable *via* advanced 3D printing or micro/nano fabricated structures, are required. Another opportunity lies in moving from deterministic to stochastic geometries. The TME's architecture is not perfectly ordered, so future models should incorporate controlled but non-uniform geometries that better translate the fibrous ECM network found in the TME, allowing for the study of immune cell-migration in a geometry that resembles what we see *in vivo*. Geometries like this have been fabricated and used previously and could be incorporated into these types of models.^43–47^

Finally, the field must move from decoupling to re-coupling variables. This is the essence of a system design challenge: to build multi-variable systems that, for example, test the effect of physical confinement, plus stiffness, plus a weak opposing chemokine gradient, all within one chip. Only by embracing this complexity in a controlled, engineerable platform can we begin to understand the non-linear emergent behaviours that govern T-cell failure in solid tumours *in vivo*.

## 5. Bridging the gap: engineering infiltration-competent CAR T-cells

The primary value of isolating physical confinement is not just in modelling the TME, but in creating a robust design-build-test-learn (DBTL) cycle to engineer infiltration-competent CAR T-cells. The failure of conventional *in vitro* assays to predict *in vivo* failure represents a critical translational gap. We propose that the design of microfluidic platforms incorporating physical confinement is the missing test phase in this cycle, providing a high-throughput, physiologically relevant assay to screen for infiltration, not just activation or cytotoxic efficacy.

This approach allows us to move beyond screening for whether a CAR T-cell can kill, and ask if it can arrive at its target, and, critically, in what state it arrives. This is a biophysical problem that demands a well-designed and fabricated solution. These confinement-focused platforms can be used to validate novel engineering targets and combination strategies.

Apart from studying how CAR T-cells migrate and their metabolic state, these platforms can also be used to discover insights into what to engineer in the T-cell itself. For instance, given the evidence that the nucleus is the primary bottleneck,^37^ platforms can test CAR T-cells engineered to be “softer” or more “deformable”, perhaps *via* transient modulation of nuclear lamina proteins (*e.g.*, lamin A/C).^13^ Another way can be engineering the knockout of GEF-H1, a Rho guanine exchange factor that increases Rho activity upon microtubules destabilization, which has been shown to significantly enhance T-cell migration in complex 3D environments.^13^ Concurrently, given the high metabolic cost of migration,^16^ these platforms also give a space to test cells engineered with augmented mitochondrial performance or superior metabolic programs to resist exhaustion. In addition to discrete genetic edits, there is an emerging potential to reprogram T-cells to adopt innate-like functional programs, creating a hybrid cell source that combines the antigen specificity of T-cells with the superior infiltration and metabolic resilience typical of NK cells.^48,49^ As the field moves toward next-generation therapies, precision-engineered microfluidic environments will serve as essential pre-clinical testbeds to validate the migration dynamics and efficacy of these cells with joint innate and adaptive characteristics before they reach clinical application.

Alternatively, the platforms proposed paired with the right materials are ideal for testing TME-modifying co-therapies. Engineering the TME could be another path to tackle migration challenges. For example, the combination of cancer-targeted and stroma-targeting CAR T-cells directed against specific fibronectin (FN) domains could facilitate infiltration of the tumour by actively dismantling the physical barrier built by the tumour stroma.^50^ Another alternative is using enzyme-secreting CAR T-cells, such as those that secrete heparanase^51^ or hyaluronidase^52^ to facilitate infiltration. This could also include the pre-treatment or co-administration of enzymatic agents (*e.g.*, collagenase) to degrade the ECM, or pharmacological agents like TGF-β inhibitors, which can reduce fibroblast-mediated ECM deposition and stimulate T-cell activity.^53^ Such models could be quantitatively assessed in these specifically designed environments.

A confinement-based microdevice is the ideal testbed to quantify the engineering trade-offs of these strategies. Using high-content analysis, we can simultaneously measure migration speed through defined constrictions against metrics of cell health and damage or metabolic state, such as mitochondrial activity, nuclear deformation and reactive oxygen species accumulation. This allows us to move beyond a binary assay and into a quantitative design space, identifying strategies that enhance migration without fatally compromising the cell's genomic integrity or metabolic reserves. This DBTL cycle, built upon a foundation of confinement-focused microfluidics, is the most direct path to engineering CAR-T cells that is truly appropriate for solid tumours.

## 6. Conclusions

The efficacy of adoptive cell therapies in solid tumours is fundamentally tied to a physical engineering challenge: infiltration. In this perspective, we have argued that physical confinement by the TME's geometric architecture is a primary, underappreciated, and restrictive barrier that modulates other biochemical and metabolic challenges. The solution to this physical problem lies in design. Microfluidic systems are not just passive models; they are precision-engineered instruments that allow us to deconstruct the TME fortress, isolate the variable of confinement, and quantify its profound impact on T-cell efficacy. By placing physical confinement at the centre of our design strategy, both for our *in vitro* testbeds and for the CAR T-cells themselves, we can systematically test new hypotheses and begin to engineer therapies that can finally conquer solid tumours.

While understanding and engineering for confinement is, in our view, the most pressing initial task, it is only the first step. To further enhance biological relevance, these confinement models must eventually be integrated with other key TME barriers. This includes the incorporation of endothelialized vascular networks to model the full extravasation-to-infiltration cascade,^4,17^ the inclusion of patient-derived organoids to recapitulate native tumour architecture,^7^ and the development of multi-parametric readouts to correlate migration dynamics with real-time cell state. By systematically building this complexity upon a robust, well-understood biophysical foundation, we can truly bridge the *in vitro–in vivo* gap and engineer the next generation of CAR-T cells.

## Author contributions

Valeria Gonzalez Abrego: conceptualization; methodology; investigation; resources; data curation; formal analysis; writing – original draft preparation; visualization. Matthew Chin: resources; writing – review & editing; supervision. Marc-Olivier Coppens: resources; writing – review & editing; supervision; project administration; funding acquisition.

## Conflicts of interest

There are no conflicts to declare.

## Data Availability

No primary research results, software or code have been included, and no new data were generated or analysed as part of this review.
